# Dietary diversity feeding practice and its associated factors among children age 6–23 months in Ethiopia from 2011 up to 2018: a systematic review and meta-analysis

**DOI:** 10.1186/s13052-018-0567-9

**Published:** 2018-09-17

**Authors:** Habtamu Temesgen, Ayenew Negesse, Wubetu Woyraw, Nakachew Mekonnen

**Affiliations:** 1 Department of Human Nutrition and Food Sciences, College of Health Science, Debre Markos University, P.O. Box 269, Debre Markos, Ethiopia; 2 Department of Public Health, College of Health Science Debre Markos University, P.O. Box 269, Debre Markos, Ethiopia

**Keywords:** Dietary diversity, Pooled prevalence, Age 6–23 months, Ethiopia

## Abstract

**Background:**

Globally Less than one fourth of children aged 6–23 months get the recommended minimum dietary diversity feeding practice. Despite this issue is common in Ethiopia, fragmented and inconsistent findings were found. Therefore the main objective of this meta-analysis was to estimate the pooled prevalence of dietary diversity feeding practice and to identify its associated factors among children aged 6–23 months in Ethiopia.

**Methods:**

The Preferred Reporting Items for Systematic Reviews and Meta-Analyses guideline was followed. Articles were systematically searched through PubMed, Google scholar, Google, Hinari and Cochrane library. Newcastle-Ottawa Scale adapted for cross-sectional studies quality assessment tool was used to assess the quality of each study. A total of 14 studies were extracted and analyzed using STATA 14. Random effect model was used to estimate the pooled prevalence; whereas subgroup analysis and meta-regression was performed to identify the probable source of heterogeneity. Both egger’s and begg’s test were used to check publication bias. Furthermore, the effect between associated factor variables, and dietary diversity feeding practices were examined.

**Results:**

A total of 154 studies were retrieved and 14 studies were included in meta-analysis. The Meta analysis result showed that the pooled prevalence of dietary feeding practice among children age 6–23 months in Ethiopia was 23.25% with considerable heterogeneity (I^2^ = 98.8, *p* = 0.00). In the subgroup analysis, the lowest prevalence was observed in Amhara region (12.58%). Home delivery OR: 0.63, antenatal care follow up OR: 1.80, postnatal care visit OR: 2.61, mothers decision making status OR: 1.65, mothers media exposure status OR: 2.79 and being urban residence OR: 2.18 (1.26, 3.77) were significant factors for minimum dietary diversity feeding practice in Ethiopia.

**Conclusions:**

The pooled prevalence of dietary diversity feeding practice among children aged 6–23 months in Ethiopia was low. Place of delivery, post natal care, antenatal care service, mothers decision making status, mothers media exposure status and being urban residence were found to be the significant factors.

## Background

Proper infant and child feeding practice is fundamental for optimal child growth and development [1]. Suboptimal infant and young child feeding practices contributes to irreversible outcomes of nutritional problems. It is highly prevalent in low and middle-income countries resulting in considerable increase in mortality and overall disease burden [2–5]. Globally, out of 10.9 million under-5 year deaths that occur, malnutrition is directly or indirectly responsible for 60% of death. More than 3.4 million under-5 year children die each year due to inappropriate feeding Practices. Of which, two-thirds of these deaths are associated with inappropriate feeding practices during the first 2 years of life [6, 7].

Infant and young child feeding during the first two years of life is a key area to improve child survival, healthy, growth and development. Even though diversified food for children aged 6–23 months important, only less than a fourth of them globally get recommended diversified diet [8, 9]. Appropriate dietary diversity feeding practice during this critical periods can decrease morbidity, mortality, risk of chronic disease, and developmental delays [8].

Dietary diversity is a quantitative number of food items or groups used as a method of determining variety and nutrient adequacy of diets for an individual [10]. The number of different food groups consumed over a given reference period, has been identified as a potentially useful indicator for nutrient adequacy [11]. Nutrient rich foods from diverse diets are important elements in child feeding practices that support dietary needs and adequate growth during their early years of life especially within two years of life [12]. Hence, nutrient adequacy of the diet consumed by the individuals depends on the individual dietary diversity score [13, 14]. According to World health organization children aged 6–23 month should consume foods among the seven food groups [(1).Grains, roots and tubers; (2). Legumes and nuts; (3).Dairy products; (4). Flesh foods (meats/fish/poultry); (5).Eggs; (6). Vitamin A-rich fruits and vegetables; and (7).Other fruits and vegetable]. Breast feed child must feed four and above and non-breast feed child should feed milk and milk products in addition to the four food groups [11, 15].

In Ethiopia different activities were conducted to increase the nutritional status of children and currently Federal Ministry of Health in collaboration with other sectors had developed national nutrition plan that includes complementary feeding practice for children aged 6–23 months [16].

Even though different activities were conducted to increase dietary diversity feeding practice in Ethiopia, the problem is still high and varied from area to area. In Ethiopia, different independent and fragmented studies have been conducted to assess the magnitude of dietary diversity and its associated factors of children aged 6–23 months. These separate studies indicated that the magnitude of minimum dietary diversity of children aged 6–23 months in Ethiopia were from 7 to 59.9% [17–31]. From the reports on these studies, there were great variation and inconsistency related to the diversified dietary feeding practice and its associated factors throughout the country [17, 31]. The most common factors reported by Ethiopian studies were post natal care, antenatal care, place of delivery, residence, decision making status,place of residence and media exposure of the mothers [18, 20, 21, 23, 25–27, 29, 30]. The reasons for variations in the magnitude of dietary diversity feeding practice and its associated factors of Ethiopian children aged 6–23 months have not yet been investigated. Therefore, the main objective of this systematic review and meta-analysis was to estimate the pooled prevalence of minimum dietary diversity feeding practice and to identify its predictors among children aged 6–23 months in Ethiopia.

The findings of this study will be an input to policy makers and program planners of Ethiopian government relevant bodies and will help to design appropriate interventions to improve diversified dietary feeding practice of children aged 6–23 months, and help to intervene important factors. This review will also have greatly help future researchers of related topics.

## Methods

### Searching strategies

This systemic review and meta-analysis was designed to estimate the pooled prevalence of dietary diversity among children age 6–23 months and identify significant associated factors for dietary diversity in Ethiopia. This systematic review and meta-analysis was conducted by reviewing different literatures. Both Published and unpublished research reports on the prevalence and associated factors of dietary diversity feeding practice among children aged 6–23 months in Ethiopia were considered. But we found only those published researches and it is difficult to find the unpublished researches. Our search for the published articles were restricted by age (children 6–23 months age) and by country (the studies conducted only in Ethiopia).The international databases, including PubMed, Google scholar, Hinari, direct Google search and Cochrane library were systematically searched. The search was carried out using the following MESH TERMS “prevalence”, “minimum dietary diversity among children”, “dietary diversity score”, “factors for dietary diversity” and “Ethiopia”. The search terms were used separately and in Combination using “OR” or “AND”. The search was conducted from February, 2018 to March 30, 2018. All published articles up to March 30, 2018 were included in the systematic review. Preferred Reporting Items for Systematic Reviews and Meta-Analyses (PRISMA) guideline was used during the systematic review [32].

### Eligibility criteria (inclusion, exclusion criteria and outcome measurement)

#### Inclusion criteria

Study area: Only studies conducted in Ethiopia.

Publication condition: articles published in peer reviewed journals.

Study design: all original studies that report the prevalence of minimum dietary diversity and its associated factors among children aged 6–23 months in Ethiopia.

Language: Articles published in English language.

#### Exclusion criteria

Studies difficult to access full text, not in English language and studies which didn’t report specific outcomes were excluded.

### Outcome measurement

The primary outcome of this study was prevalence of minimum dietary diversity feeding practice among children aged 6–23 months. The second outcome of the study was the most frequent associated factors of dietary diversity feeding practices.

### Data abstraction

Data were extracted by two authors (HT and AN) using a standardized data extraction spread sheet. The data extraction format includes primary author, publication year, region where the study was conducted, sample size, prevalence of minimum dietary diversity and factors (postnatal care, place of delivery, media exposure status, maternal decision making and residence with their cross tabulation to dietary diversity (a, b, c and d)).

### Quality assessment

Newcastle-Ottawa Scale adapted for cross-sectional studies quality assessment tool was used to assess the quality of each study [33]. The tool has mainly three sections the first section graded from five stars and mainly focused on the methodological quality of each study. The second section of the tool deals with the comparability of the study. The last section deals with the outcome and statistical analysis of each original study. Two independent reviewers (AN and WW) critically evaluated each individual papers. Disagreements between those reviewers were solved by discussion. If not, third reviewer (NM) was involved to resolve inconsistencies in between the two independent reviewers. Studies which score 50% and above were included in the final review and analysis. The Database search results were combined and duplicate articles were removed manually using Endnote (version X7.2).

### Statistical analysis

The extracted data were entered into computer using excel sheet and imported to STATA 14 for analysis. Heterogeneity among reported prevalence was assessed by using the inverse variance (I^2^) with Cochran Q statistic at 25%, 50% and 75% as low, moderate and considerable heterogeneity respectively with *p*-value less than 0.05 [34]. Random effects meta-analysis model was used to estimate the pooled prevalence of dietary diversity. Forest plot was also used to visualize the presence of heterogeneity subjectively. Possible differences between studies were explored by sub-group analyses and Meta regression. The finding was presented using forest plot with respective odds ratio and 95% confidence intervals. Evidence of publication bias was assessed using both egger’s and begg’s test with *p*-value of less than 0.05 as a cut of point to declare the presence of publication bias [35, 36]. For the second outcomes, pooled odds ratios with 95% CI for each factor were used to determine the association between dietary diversity and its predictors (ANC, PNC, place of delivery, decision making status, media exposure and place of residence).

## Result

### Search results

**Fig. 1:**
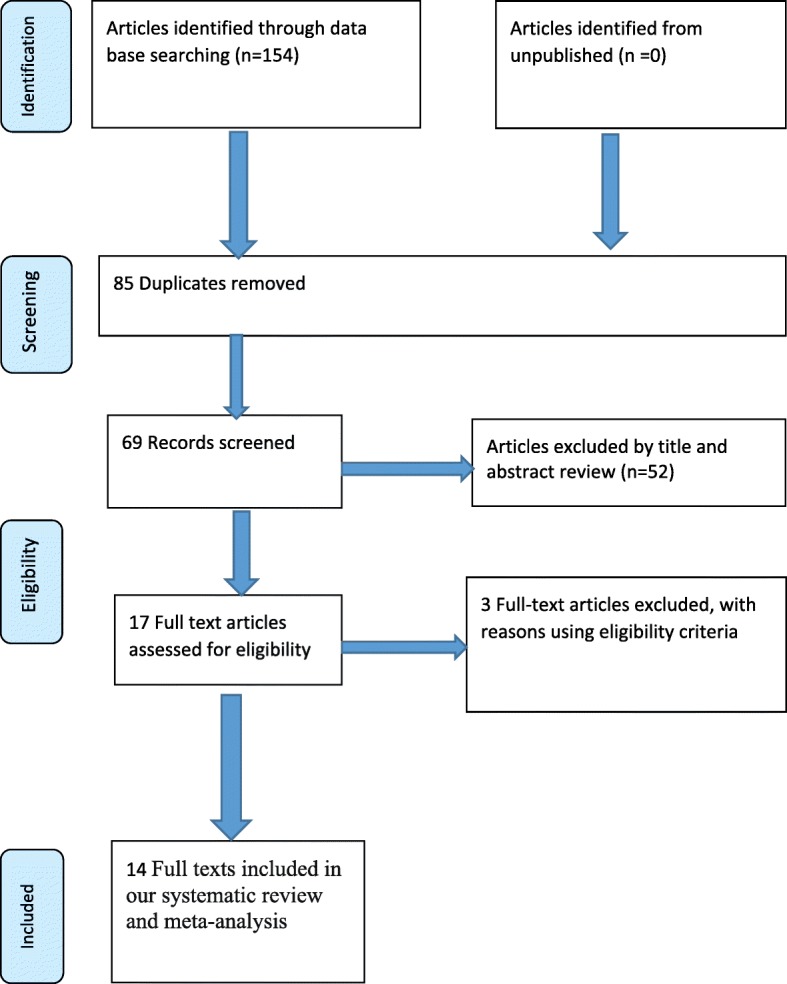
PRISMA flow diagram of included studies on dietary diversity feeding practice in Ethiopia children

A total of 154 studies were retrieved concerning the magnitude of dietary diversity and its factors which were conducted at different levels in Ethiopia using different electronic data bases as described in the search strategy. From these articles 85 articles were excluded due to duplication. From the remaining 69 articles, 52 articles were non relevant and excluded after reading of titles and abstracts. Finally 17 full text articles were accessed for eligibility criteria. Based on the pre-defined criteria and after critical appraisal, only 14 articles were included for the final analysis (Fig. 1).

#### Characteristics of the original articles

There were 14 original studies included for systematic review and meta-analysis from 2011 up to 2018. In the current meta-analysis, 13,531 mothers who had children’s aged 6–23 months were involved to estimate the pooled prevalence of dietary diversity among children aged 6–23 months. The highest prevalence of dietary diversity was reported from a study done in Addis Ababa `town (59.9%) [31] and the lowest prevalence (7%) was reported from a study done in food insecure area of south Wollo, Amhara regional state [17]. In this systematic review and meta-analysis, five regions, one city administration and one nationwide study were included. One study from Addis Ababa [31], one nationwide study [18], one from Afar region [27], three studies were from Oromya region [19, 25, 29], three studies were from Southern Nations, Nationalities and Peoples’ region [23, 24, 28] and five studies were from Amhara region [17, 20, 21, 26, 30]. The design of all the studies were cross-sectional study design. Only one study was conducted at facility, facility based cross-sectional [31]. But all others were community based cross sectional studies. Three studies were conducted in urban areas [21, 25, 31] while all the rest were conducted in urban and rural settings. Among these one study used secondary data for the analysis [18]. The response rate of each original study ranged from 95.8 to 100%. All articles that were included were published in reliable journal in the time range of 2013 up to 2018 (Table 1).

**Table 1:** Characteristics of studies included in the systematic review and meta-analysis to estimate the pooled prevalence of dietary diversity among children age 6–23 months in Ethiopia from 2011 up to 2018

Authors	Region	Study year	Publication year	Study design	Sample size	Actual Sample used	Prevalence (at 95% CI)
Tegegne et al. [29]	Oromya	2016	2017	cross-sectional	836	801	28.5(25.37,31.63)
Solomon et al. [31]	Addis Ababa	2016	2017	cross-sectional	365	352	59.9(54.78,65.02)
Mekonnen et al. [28]	SPNN	2015	2017	cross-sectional	623	611	27.3(23.77,30.83)
Temesgen et al. [30]	Amhara	2016	2018	cross-sectional	740	736	13(10.57,15.43)
Beyene et al. [21]	Amhara	2014	2015	cross-sectional	925	920	12.6(10.46,14.74)
Belew et al. [20]	Amhara	2016	2017	cross-sectional	1034	1034	17(14.71,19.29)
Aemro et al. [18]	Ethiopia	2011	2013	cross-sectional	2836	2836	10.8(9.66,11.94)
Kumera et al. [26]	Amhara	2016	2018	cross-sectional	967	955	13.6(11.43,15.77)
Agize et al. [19]	Oromya	2014	2016	cross-sectional	730	700	16(13.28,18.72)
Gatahun, et al. [24]	SPNN	2014	2015	cross-sectional	759	562	23.3(19.80,26.80)
Getu Gamo Sagaro [23]	SPNN	2016	2017	cross-sectional	944	939	43.2(40.03,46.37)
Gebremedhin et al. [17]	Amhara	2014	2016	cross-sectional	2080	2080	7(5.90,8.10)
Gebremichael et al. [25]	Oromya	2016	2017	Cross-sectional	647	635	25.2(21.82,28.58)
Legesse et al. [27]	Afar	2015	2017	cross-sectional	370	370	30.8(26.10,35.50)

A total of six studies reported that antenatal care follow up for the index child were significant associated factors for dietary diversity feeding of children [18, 23–25, 27, 28].On the other hand, only three studies were reported that mothers involved on decision making [18, 21, 30]; Eight studies reported place of delivery [18, 20, 21, 23–25, 28, 30], and five studies reported residence [18, 20, 21, 24, 26]. Four studies reported postnatal care service for the index child [20, 24, 29, 30] and six studies reported media exposure status of the household [18, 21, 24, 26, 29, 30] which were significant associated factors of dietary diversity feeding practices of children aged 6–23 months in Ethiopia separately.

### Meta-analysis

**Fig. 2:**
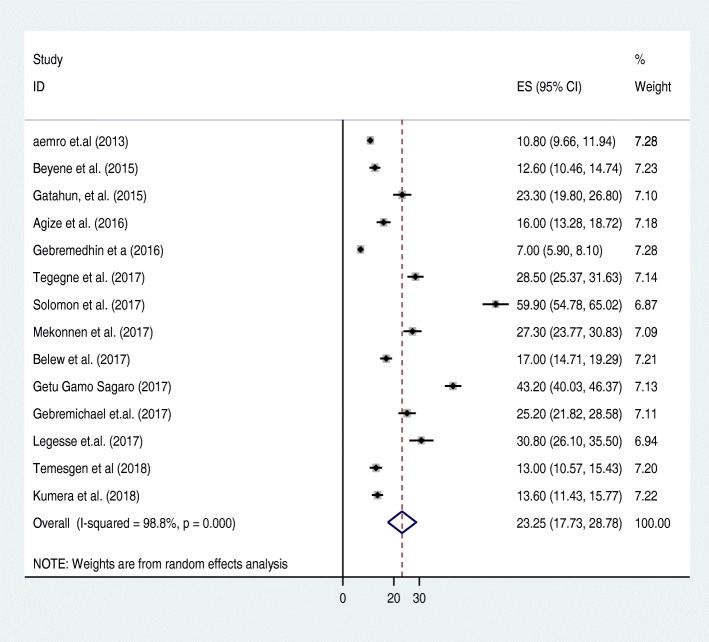
Forest plot for pooled prevalence of dietary diversity of children, 6–23 months in Ethiopia

The results of 14 studies showed that, the pooled prevalence of dietary diversity feeding practice among children aged 6–23 months in Ethiopia was 23.25% (CI: 17.73, 28.78) after we calculated the standard error of prevalence for each original article. Considerable heterogeneity was detected across the studies (I^2^ = 98.8, *p* value = 0.00). Due to the presence of considerable heterogeneity across the studies, random effect analysis model was employed to estimate the pooled prevalence of dietary diversity feeding practice of children aged 6–23 months in Ethiopia (Fig. 2).

#### Sub group analysis

Because of heterogeneity (Fig. 2), we performed subgroup analysis based on region, years of study and study setting to identify the possible source of heterogeneity across the studies. But the subgroup analysis result reveals that the source of heterogeneity was not due to region, time of the study and place of the study area disparities. The lowest pooled prevalence of dietary diversity among children aged 6–23 months was in Amhara region [12.58%(CI:8.61,16.55)] and the highest was at others regions [33.76%(CI:4.80,62.71)] followed by SPNN [31.29(CI:19:00,43.57)] (Table 2). The prevalence of dietary diversified feeding after 2015 was 28.49 (CI: 18.38, 38.59) (Table2.). Moreover, the studies conducted at urban setting have high dietary diversity feeding practice (Table2). Publication bias as the source of heterogeneity was also checked using both begg’s and egger’s test. The result of Begg and Egger tests were statistically significant for estimating the Prevalence dietary diversity (*p* = 0.00) and (*p* = 0.001) respectively.

**Table 2:** Sub group analysis of the pooled prevalence dietary diversity feeding practice among children aged 6–23 months in Ethiopia from 2011 up to 2018

Sub group by		Number of studies included	Prevalence (95% CI)	Heterogeneity statistics	*P* value	I^2^	Tau-squared
Region	Amhara	5	12.58(CI:8.61,16.55)	85.62	0.00	98.8%	19.36
	Oromia	3	23.20(CI:15.43,30.96)	38.60	0.00	94.8%	44.65
	SNNP	3	31.29(CI:19:00,43.57)	78.60	0.00	97.5%	114.91
	^a^Others	3	33.76(CI:4.80,62.71)	387.87	0.00	99.5%	650.62
Time of study	After year 2015	7	28.490 (18.38, 38.59)	530.66	0.00	98.9%	183.43
	Within and before 2015	7	23.254 (17.73, 28.78)	262.8	0.00	97.7%	43.74
Place (study setting)	Towns	3	32.46(9.22, 55.69)	295.20	0.000	99.3%	418.14
	Rural and town	11	20.84(15.28, 26.39)	751.64	0.000	98.7%	86.47

^a^Addis Ababa, afar, Ethiopia

#### Meta regression

The Subgroup analysis didn’t display the source of heterogeneity. Therefore, Meta regression was also undertaken by considering both continuous and categorical data. Region, sample size, years of the study and study area (town/mixed) were considered in the meta-regression. But the variables that were included in the Meta regression revealed that heterogeneity in the prevalence of dietary diversity of children in Ethiopia was not associated with region, sample size variation, Time of the study and study area (town/mixed) (Table 3).

**Table 3:** Meta regression to identify source of heterogeneity for the prevalence of dietary diversity among children age 6–23 months in Ethiopia, 2011 up to 2018

Variables		Coefficients	*p*-value
Region	Oromia	10.59	0.27
	SNNP	20.87	0.05
	Others	18.65	0.07
Sample size	sample included in the analysis	−.0110771	0.05
Time of the study	After 2015	10.31065	0.186
Study area	Town	11.43705	0.233

### Factors associated with dietary diversity feeding practice of children age 6–23 months in Ethiopia

Considering the total of 11 [18, 20, 21, 23–26, 28–31] studies with reported factors, we tried to estimate the overall pooled effect size of different factors reported repeatedly that affect the dietary diversity of children age 6–23 months age in Ethiopia. We have tested for the associations of place of delivery [18, 20, 21, 23–25, 28, 30], antenatal care for index child [18, 23–25, 28], decision making status of the mother [18, 21, 30], media exposure status [18, 21, 24, 26, 29, 30], residence [18, 20, 21, 24, 26] and post natal care service [20, 24, 29, 30] with dietary diversity feeding practice of children.

**Fig. 3:**
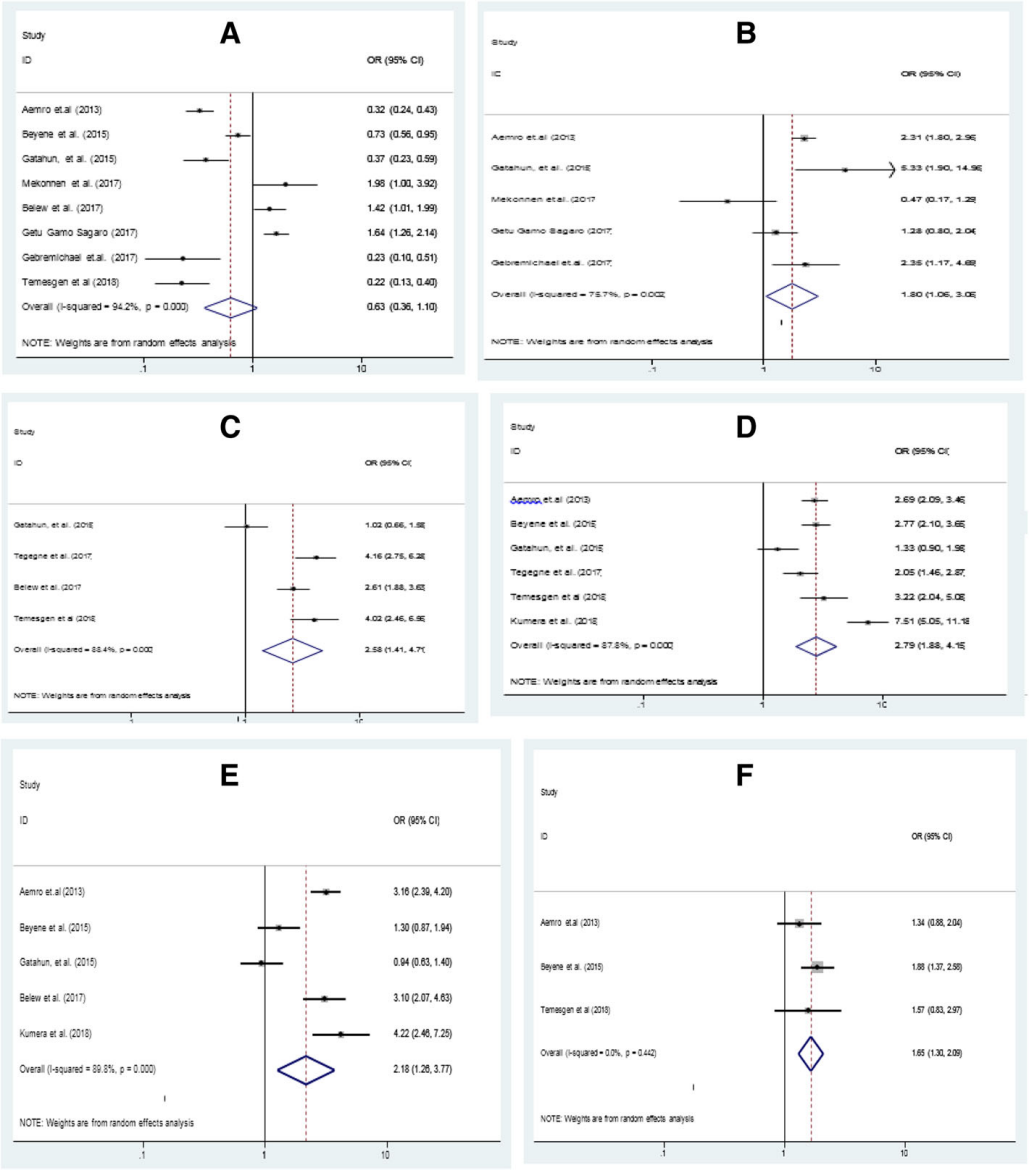
Forest plot showing pooled odds ratio of the associated factors for dietary diversity. (**a** Place of delivery, **b** Antenatal care, **c** Postnatal care, **d** Exposed to media, **e** Urban residence and **f** Mother involved in decision making)

Among eleven studies, eight of them revealed that place of delivery was significantly associated with dietary diversity feeding practice among children aged 6–23 months, odds ratio 0.63 (95% CI .36,1.10)(Fig. 3a). This indicates that, mothers who delivered the index child at home were 63% times less likely fed diversified diet than who were attended at health institutions. Five studies also indicated that presence of ANC follow up during pregnancy was significantly associated with dietary diversity. Those mothers who had ANC follow up for index child were 1.8 times more likely to get diversified diet than counterparts,odds ratio 1.80 (95% CI: 1.06,3.05) (Fig. 3b). Moreover, the results of five studies showed that presence of post natal care increases diversified feeding 2.61 times more than their counterparts, odds ratio 2.61(95% CI:2.15,3.18) (Fig. 3c).

Furthermore, results from six studies of meta-analyses showed that, media exposure of the household was significantly associated for dietary diversity feeding practice of children aged 6–23 months in Ethiopia. Households who were exposed to media source were 2.79 times more likely to get diversified diet than who were not exposed, odds ratio 2.79 (95%CI: 1.88, 4.15) (Fig. 3d) Similarly, being urban residence (result from five studies) showed 2.18 times more likely fed diversified diet than rural residences, odds ratio 2.18 (1.26, 3.77) (Fig. 3e). Finally, the Meta analysis result of effect size from three studies showed that, mothers involved on decision making in the household were 1.65 times more likely feed diversified diet for their child than counterparts, odds ratio 1.65(95% CI: 1.30, 2.09) (Fig. 3f).

## Discussion

The main objectives of this meta-analysis were to estimate the pooled prevalence of dietary diversity feeding practice and its associated factors of children aged 6–23 months in Ethiopia from 2011 up to 2018. As far as our source and knowledge is concerned, this meta-analysis is the first in Ethiopia to estimate the pooled prevalence and associated factors of dietary diversity feeding practice.

According to this meta-analysis the overall pooled prevalence of dietary diversity feeding practices among children age 6–23 months in Ethiopia that meets the minimum dietary diversity score (four and above food items from the seven food groups) was 23.25% (CI: 17.73 28.78). This Indicates that in Ethiopia, almost one fourth of the child had got at least four food groups from the seven food groups. It means that a high likelihood of consuming at least one animal-source food and at least one fruit or vegetable, in addition to a staple food [11].

Even though related study (meta-analysis) on this specific research question has not yet been conducted in Ethiopia, the prevalence reported in the present assessment is higher than the national survey conducted in Ethiopia (10.8%, 7.8%, and 4%) [18, 37, 38]. The possible Justification for this variation could be due to the study period and study area. However, the prevalence of dietary diversity of child was lower than the study reported in Bangladesh (48.4%) and Vietnam (83.2%) [37], Ghana (47·7%) [39], Nepalese(30.4%) [40], Zambia (37%) [41] and Sri Lanka (71%) [42]. This disparity may be due to the differences in study area and periods, socio economic status of the country, method of study and policies related to infant and young child feeding.

The subgroup analysis of this study showed that the pooled prevalence of dietary diversity feeding practices among children aged 6–23 months is significantly across regions of the Ethiopia. The highest prevalence of dietary diversity feeding of Ethiopian children aged 6–23 months were observed in other regions (33.76%) followed by SPNN (31.29%) while the lowest prevalence was observed in Amhara region with the prevalence of 12.58%.The possible reason for this variations are due to the difference in study area and may other reasons like livelihood practice variations. The highest prevalence was observed in urban setting for example in Addis Ababa (59.9%) [31] during the study periods. This is also supported by the Ethiopian demographic health survey 2016 report indicates the highest prevalence of stunting observed in Amhara regions which occurred due to chronic infant and young child feeding problems, directly related to low diversified dietary feeding based on the recommended criteria [43]. On the other had studies that were conducted after 2015 indicated relatively higher prevalence of dietary diversity feeding practice among children aged 6–23 months in Ethiopia. This may be due to different activities and programs that have been undertaken to increase the infant and young child feeding practice after the end of millennium development goals. More-over, the study that was conducted at urban setting had higher dietary diversity feeding prevalence than rural settings.

The current meta-analysis study also aimed that to identify the associated factors of diversified dietary feeding practice among children aged 6–23 months in Ethiopia. In the current meta-analysis, health institution delivery of index child, presence of ANC follow up, PNC visit, media exposure of the household, maternal decision making status at the house hold and being urban residence were found to be significant factors associated with minimum dietary diversity feeding practice. Mothers who delivered the index child at home were 63% times less likely to feed diversified diet for their child than those delivery was at health institution. Mothers that had ANC follow up and postnatal care visit for the index child were 1.8 and 2.61 times more likely to get diversified diet than counterparts respectively. The possible reasons for the association may be due to the strong counseling during perinatal periods and awareness creation about complementary feeding practice and the health care providers’ demonstration at each health institution how to prepare diversified complementary food preparation for their children during ANC, PNC and during vaccination periods.

Furthermore, in this meta-analysis we noticed that those mothers who had satisfactory media exposure showed that, 2.79 times more likely feed their children aged 6–23 months than those with limited exposure. This indicates Media can be used as an effective means of promoting infant and young child feeding practice. This is in line with the study conducted in Ethiopia [18]. This study also reported that, mothers involved in decision making were positively associated with recommended minimum dietary diversity feeding practices in Ethiopia. The possible reason could be, in Ethiopia mostly child feeding is the responsibility of mothers. Therefore involvement of mothers on their household matters can empower mothers to feed diversified diet for their child. On the other hand children born from mothers that lived in urban areas were2.18 times more likely to get minimum dietary diversity than rural residences. This is in line with the study conducted in Nepalese [40]. The probable justification is that mothers who live in the urban area have relatively good awareness on infant and young child feeding practices. Because urban mothers have different exposure of information which stimulates the importance of dietary diversified feeding practices as compared to rural mothers. Urban mothers have access of health service and get adequate counseling about complementary feeding practice at every contact until child age 24 months.

### Limitations of the study

The first limitations of the study was only English articles were considered for the analysis. All studies included in this review were cross sectional in nature and therefore the outcome variable might be affected by other confounding variables. Moreover, this meta-analysis represented only studies reported from five regions of the country and one from nationwide EDHS study.

## Conclusion

This systematic review and meta-analysis indicates that, the pooled prevalence of recommended dietary diversity feeding practice among children aged 6–23 months in Ethiopia was low, almost one fourth of children got the minimum recommended dietary diversity in Ethiopia. Institutional delivery, post natal care follow up service, antenatal care service, mother involvement on decision making, mothers being exposed to media and being urban residence were found to be the factors of minimum dietary diversity feeding practice among children aged 6–23 months in Ethiopia. Therefore, based on our outcomes, we strongly recommend that efforts should be strengthen to increase the proportion of children who get recommended dietary diversity diets. The government of Ethiopia should strength maternal health service utilization and empowerment of women’s to participate on decision making. More emphasis should be given for rural women. In addition these, collaboration of different stakeholders for awareness creation about dietary diversity feeding practice through different means of communication should be strengthened.

## Abbreviations

AN: Ayenew negesse
ANC: Antenatal care
HT: Habtamu temesgen
NM: Nakachew Mekonnen
OR: Odds ratio
PNC: Post natal care
PRISMA: Preferred reporting items for systematic reviews and meta-analyses
WW: Wubetu woyiraw

## References

[1] Lutter C. K., Daelmans B. M. E. G., de Onis M., Kothari M. T., Ruel M. T., Arimond M., Deitchler M., Dewey K. G., Blossner M., Borghi E. (2011). Undernutrition, Poor Feeding Practices, and Low Coverage of Key Nutrition Interventions. PEDIATRICS.

[2] Organization WH (2009). Infant and young child feeding: model chapter for textbooks for medical students and allied health professionals.

[3] Black RE, Allen LH, Bhutta ZA, Caulfield LE, De Onis M, Ezzati M, Mathers C, Rivera J (2008). Maternal, group CUS: maternal and child undernutrition: global and regional exposures and health consequences. Lancet.

[4] Onyango AW, Borghi E, de Onis M, del Carmen Casanovas M, Garza C (2014). Complementary feeding and attained linear growth among 6–23-month-old children. Public Health Nutr.

[5] Arimond M, Ruel MT (2004). Dietary diversity is associated with child nutritional status: evidence from 11 demographic and health surveys. J Nutr.

[6] Kumera G, Tsedal E, Ayana M (2018). Dietary diversity and associated factors among children of orthodox Christian mothers/caregivers during the fasting season in Dejen District, north West Ethiopia. Nutrition & metabolism.

[7] Nutrition IC (2013). The achievable imperative for global progress.

[8] WHO (2017). Infant and young child feeding. WHO fact sheet.

[9] WHO: Infant and young child feeding. WHO fact sheet 2015 fact sheet 342.

[10] Sealey-Potts C, Potts A (2014). An assessment of dietary diversity and nutritional status of preschool children. Austin Journal of Nutrition and Food Sciences.

[11] Daelmans B, Dewey K, Arimond M (2009). New and updated indicators for assessing infant and young child feeding. Food Nutr Bull.

[12] Dewey K (2002). Guiding principles for complementary feeding of the breastfed child.

[13] Nutrition F (2011). Consumer protection division. Guidelines for measuring household and individual dietary diversity Rome.

[14] Aemro M, Mesele M, Birhanu Z, Atenafu A. Dietary diversity and meal frequency practices among infant and young children aged 6–23 months in Ethiopia: a secondary analysis of Ethiopian demographic and health survey 2011. Journal of nutrition and metabolism. 2013;2013. 10.1155/2013/782931 PMC3878383 24455218

[15] Organization WH (2010). Indicators for assessing infant and young child feeding practices part 3: country profiles.

[16] Ethiopia Fdro: National nutrition program. 2016-2020.

[17] Gebremedhin S, Baye K, Bekele T, Tharaney M, Asrat Y, Abebe Y, Reta N: Predictors of dietary diversity in children ages 6 to 23 mo in largely food-insecure area of south Wollo, Ethiopia. Nutrition (Burbank, Los Angeles County, Calif) 2017, 33:163–168. 10.1016/j.nut.2016.06.002 27499206

[18] Aemro M, Mesele M, Birhanu Z, Atenafu A (2013). Dietary diversity and meal frequency practices among infant and young children aged 6-23 months in Ethiopia: a secondary analysis of Ethiopian demographic and health survey 2011. Journal of nutrition and metabolism.

[19] Agize A, Jara D, Dejenu G (2017). Level of knowledge and practice of mothers on minimum dietary diversity practices and associated factors for 6-23-month-old children in Adea Woreda, Oromia. Ethiopia BioMed research international.

[20] Belew AK, Ali BM, Abebe Z, Dachew BA (2017). Dietary diversity and meal frequency among infant and young children: a community based study. Ital J Pediatr.

[21] Beyene M, Worku AG, Wassie MM (2015). Dietary diversity, meal frequency and associated factors among infant and young children in Northwest Ethiopia: a cross- sectional study. BMC Public Health.

[22] Dangura D, Gebremedhin S (2017). Dietary diversity and associated factors among children 6-23 months of age in Gorche district, southern Ethiopia: cross-sectional study. BMC Pediatr.

[23] Gamo GS, Alemayehu M. Dietary diversity and associated factors among infants and young children in Wolaita Zone, Southern Ethiopia. Sci J Clin Med. 2017;6(4):53–9.

[24] Gatahun EA, Demissie M, Abyu DM. Dietary diversity feeding practice and determinants among children aged 6-23 months in Kemba Woreda, Southern Ethiopia implication for public health intervention. J Nutr Food Sci. 2015;S13:S13003.

[25] Gebremichael B, Egata G, Assefa N (2017). Dietary diversity practice and associated factors among infants and young children in Haramaya town, Ethiopia. International Journal of Public Health Science (IJPHS).

[26] Kumera G, Tsedal E, Ayana M (2018). Dietary diversity and associated factors among children of orthodox Christian mothers/caregivers during the fasting season in Dejen District. North West Ethiopia Nutrition & metabolism.

[27] Liben M, Abuhay T, Haile Y. Factors associated with dietary diversity among children of agro pastoral households in Afar regional state. Northeastern Ethiopia, vol. 2017:5.

[28] Mekonnen TC, Workie SB, Yimer TM, Mersha WF (2017). Meal frequency and dietary diversity feeding practices among children 6-23 months of age in Wolaita Sodo town, southern Ethiopia. J Health Popul Nutr.

[29] Tegegne M, Sileshi S, Benti T, Teshome M, Woldie H (2017). Factors associated with minimal meal frequency and dietary diversity practices among infants and young children in the predominantly agrarian society of bale zone, Southeast Ethiopia: a community based cross sectional study. Archives of public health = Archives belges de sante publique.

[30] Temesgen H, Yeneabat T, Teshome M (2018). Dietary diversity and associated factors among children aged 6–23 months in Sinan Woreda, Northwest Ethiopia: a cross-sectional study. BMC nutrition.

[31] Solomon D, Aderaw Z, Tegegne TK (2017). Minimum dietary diversity and associated factors among children aged 6-23 months in Addis Ababa, Ethiopia. Int J Equity Health.

[32] Moher D, Liberati A, Tetzlaff J, Altman DG (2009). Preferred reporting items for systematic reviews and meta-analyses: the PRISMA statement. Ann Intern Med.

[33] Newcastle-Ottawa Scale customized for cross-sectional studies. Available at: https://journals.plos.org/plosone/article/file?type=supplementary&id=info:doi/10.1371/journal.pone.0147601.s001.

[34] Rücker G, Schwarzer G, Carpenter JR, Schumacher M (2008). Undue reliance on I2 in assessing heterogeneity may mislead. BMC Med Res Methodol.

[35] Begg Colin B., Mazumdar Madhuchhanda (1994). Operating Characteristics of a Rank Correlation Test for Publication Bias. Biometrics.

[36] Egger M, Smith GD, Schneider M, Minder C (1997). Bias in meta-analysis detected by a simple, graphical test. Bmj.

[37] Ali D, Saha KK, Nguyen PH, Diressie MT, Ruel MT, Menon P, Rawat R (2013). Household food insecurity is associated with higher child undernutrition in Bangladesh, Ethiopia, and Vietnam, but the effect is not mediated by child dietary diversity. J Nutr.

[38] Demographic E. Health survey: Addis Ababa. Ethiopia and Calverton, Maryland, USA: central statistics agency and ORC macro. 2011;2011.

[39] Issaka AI, Agho KE, Burns P, Page A, Dibley MJ (2015). Determinants of inadequate complementary feeding practices among children aged 6–23 months in Ghana. Public Health Nutr.

[40] Khanal V, Sauer K, Zhao Y (2013). Determinants of complementary feeding practices among Nepalese children aged 6–23 months: findings from demographic and health survey 2011. BMC Pediatr.

[41] Disha A, Rawat R, Subandoro A, Menon P (2012). Infant and young child feeding (IYCF) practices in Ethiopia and Zambia and their association with child nutrition: analysis of demographic and health survey data. Afr J Food Agric Nutr Dev.

[42] Senarath U, Agho KE, DeS A, Godakandage SS, Hazir T, Jayawickrama H, Joshi N, Kabir I, Khanam M, Patel A (2012). Comparisons of complementary feeding indicators and associated factors in children aged 6–23 months across five south Asian countries. Maternal & child nutrition.

[43] Central Statistical Agency (CSA) [Ethiopia] and ICF: Ethiopia demographic and health survey 2016. Addis Ababa, Ethiopia, and Roc kville, Maryland, USA: CSA and ICF 2016.

